# Experimental Porcine Astrovirus 4‐Associated Tracheitis and Bronchitis in Pigs

**DOI:** 10.1155/tbed/2056680

**Published:** 2026-06-12

**Authors:** Jazz Q. Stephens, Alexandra Buckley, Rachel Derscheid, Rebecca M. DuBois, Danielle J. Haley, Calvin Ko, Phillip C. Gauger, Panchan Sitthicharoenchai, Jennifer Groeltz-Thrush, Michael C. Rahe

**Affiliations:** ^1^ Department of Population Health and Pathobiology, North Carolina State University, Raleigh, North Carolina, USA, ncsu.edu; ^2^ Virus and Prion Research Unit, National Animal Disease Center, USDA, Agricultural Research Service, Ames, Iowa, USA, usda.gov; ^3^ Department of Veterinary Diagnostic and Production Animal Medicine, College of Veterinary Medicine, Iowa State University, Ames, Iowa, USA, iastate.edu; ^4^ Department of Biomolecular Engineering, University of California Santa Cruz, Santa Cruz, California, USA, ucsc.edu

**Keywords:** bronchitis, CDCD pigs, immunohistochemistry, in situ hybridization, machine learning, PoAstV4, tracheitis

## Abstract

Porcine astrovirus 4 (PoAstV4) is an emerging pathogen that has previously been associated with respiratory disease in piglets. The objective of this study was to inoculate naïve piglets with PoAstV4 and characterize the host–pathogen interaction and pathological lesions. Cesarean‐derived colostrum‐deprived (CDCD) piglets were inoculated intratracheally and intranasally with PoAstV4 PCR‐positive tissue homogenate previously screened for the presence of other primary pathogens with next‐generation sequencing (NGS). Nasal and fecal swabs for PCR identification of PoAstV4 were collected pre and postinoculation. Animals were euthanized at 5, 8, and 21 days postinoculation (DPI) and tissues were collected for histopathologic and digital image analysis. Nasal swabs were PCR positive for PoAstV4 from 2 DPI to 10 DPI. Microscopic lesions of epitheliotropic viral infection of the trachea and bronchi, including epithelial attenuation and lymphoplasmacytic infiltration, were found in inoculated pigs at 5 DPI and 8 DPI. PoAstV4 was localized to the lesions demonstrated by in situ hybridization and quantified by digital image analysis. In addition, PoAstV4‐inoculated pigs at 8 DPI had an abundance of infiltrating lymphocytes predominated by T cell lymphocytes characterized by immunohistochemistry (IHC) and quantified using digital image analysis. Both anti‐PoAstV4 IgM and IgG were detected in serum, with IgG levels first detectable at 14 DPI and increasing to the end of the study at 21 DPI. The presented findings confirm the respiratory epithelial tropism of PoAstV4 while also characterizing viral shedding and host immune response dynamics.

## 1. Introduction

Astroviruses are single‐stranded, nonenveloped, positive‐sense RNA viruses within the Astroviridae (AstV) family, which is split into two genera: *Avastrovirus* (infects birds) and *Mammastrovirus* (infects mammals). Astrovirus infection can result in a range of disease manifestations, with gastroenteritis and sporadic cases of neurologic disease reported most frequently [1–3]. Moreover, astroviruses have also been detected in the respiratory tract of people and animals with respiratory clinical symptoms or signs [4–8].

Porcine astrovirus (PoAstV) was first described in 1980, with subsequent studies describing 5 genotypes [6, 9–11]. PoAstV3 has been shown to cause polioencephalomyelitis, and PoAstV5 has been associated with enteritis [12–14]. PoAstV4 is an emerging virus that has been detected in nasal swabs from young pigs with clinical respiratory disease; however, the degree to which the virus contributed to clinical disease was unknown [6]. A retrospective evaluation to detect PoAstV4 within the respiratory tract was conducted in 117 influenza A virus (IAV) PCR‐negative cases of tracheitis and bronchitis in suckling and postweaning pigs [15, 16]. PoAstV4 was detected in the respiratory epithelium in 85 of the 117 evaluated cases, indicating a very strong association between PoAstV4 and lesions of epitheliotropic viral infection.

The objective of this study was to inoculate piglets with PoAstV4 and characterize resulting infection through the PCR quantification of PoAstV4, histopathological and digital image analysis with immunohistochemistry (IHC) and ISH, and measurement of host antibodies against the PoAstV4 capsid spike protein. Cesarean‐derived colostrum‐deprived (CDCD) piglets were used to prevent confounders such as potential maternal antibody interference or possible peri‐weaning infection with PoAstV4. Piglets were intratracheally and intranasally inoculated with a filtered PoAstV4 PCR‐positive tissue homogenate, negative for other infectious etiologies, as, to date, PoAstV4 has not been isolated.

## 2. Materials & Methods

### 2.1. Animal Study

**Figure 1:**
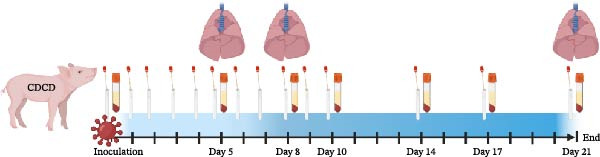
Experimental study timeline with inoculation and sampling timepoints highlighted. Figure was created with BioRender.com. Swab icon represents both fecal and nasal swabs. Blood tubes are representative of serum collection. Lungs indicate necropsy days.

All animal work was approved by the National Animal Disease Center (NADC) Institutional Animal Care and Use Committee under Protocol Number ARS‐21–0987 and ARS‐23–1138. CDCD pigs (*n* = 28) were generated at the NADC in Ames, Iowa, USA from sows purchased from a commercial swine producer. At 17 days of age, pigs were moved to an ABSL‐2 animal space, where they were randomly divided into two rooms (*n* = 17 and *n* = 11). Following a 2‐week acclimation (~30 days of age), serum, nasal swabs, and rectal swabs were collected from all pigs on 0 days postinoculation (DPI) (Figure 1). All pigs were intratracheally (3 mL) and intranasally (MAD Nasal, Teleflex, Morrisville, NC) (3 mL) inoculated with either PoAstV4 PCR‐positive homogenate, (*n* = 17, inoculated) or Dulbecco’s Modified Eagle Medium (DMEM, Gibco) (*n* = 11, negative controls). This experimental approach was similar to that used to demonstrate PoAstV3 as a cause of polioencephalomyelitis in pigs [12]. PoAstV4 inoculum homogenate originating from the lungs of a PoAstV4 confirmed clinical case from the Iowa State University Veterinary Diagnostic Laboratory (ISU VDL) was passed through a 0.22 µm filter (Corning) and screened for primary respiratory pathogens of the pig via PCR (IAV negative) and next‐generation sequencing (NGS) at the ISU VDL. The PoAstV4 cycle threshold (Ct) value for the homogenate postfiltration was 19.21, and NGS identified 166,926 reads of PoAstV4. A PoAstV4 genome, 6603 bp in length, was assembled with >99% identity to the PoAstV4 sequence identified in Padmanabhan and Hause [6] (GenBank#KU764484). No other genomic sequence segments from known or suspect primary respiratory pathogens were identified in the inoculum using NGC and bioinformatic analysis at the ISU VDL, as has previously been described [17].

Nasal and rectal swabs were collected daily from all piglets from 0 DPI to 10 DPI with subsequent sampling on 14, 17, and 21 DPI (Figure 1). Serum was collected on 5, 8, 10, 14, 17, and 21 DPI (Figure 1). At both 5 and 8 DPI, four negative controls and five inoculated pigs were euthanized. The remaining pigs were euthanized at 21 DPI. Fresh and fixed sections of proximal, middle, and distal trachea as well as the right apical bronchus, right principal bronchus, and left bronchus were collected. Sections of fresh and fixed lungs from the left cranial, right cranial, left caudal, right caudal, and accessory lobes were collected. Additionally, fixed brain, lymph nodes, nasal turbinates, ethmoid, tonsils, liver, spleen, kidney, heart, and small intestine were collected for histology.

### 2.2. PoAstV4 RT‐qPCR

All PoAstV4 RT‐qPCRs were conducted in the molecular section of the ISU VDL using standard operating procedures (SOPs). Tissues were processed into a 10% homogenate using 3 g of tissue with 27 mL of minimal essential medium (MEM; ThermoFisher) using a Genogrinder (2010 Geno/Grinder; Spex SamplePrep). Viral RNA extraction was carried out as previously described [18]. Briefly, samples were extracted using an automated magnetic particle processor system (KingFisher Apex; ThermoFisher), with a software program available through the manufacturer. The extraction was performed using a commercial extraction kit (MagMAX pathogen RNA/DNA kit; ThermoFisher) according to the manufacturer’s instructions using 100 µL of processed sample. Each sample also included an internal control to monitor PCR inhibition. Extracted nucleic acids were eluted into 90 µL of elution buffer.

The PoAstV4 reverse transcription real‐time PCR was conducted in a 20 µL reaction using 8 µL of extracted nucleic acid and 12 µL of master mix. The master mix consisted of TaqMan Fast Virus 1‐Step Mix (4x; ThermoFisher), nuclease‐free water, and the primers and probes for PoAstV4 template and internal positive control. Each plate included one positive amplification control, one negative extraction control, and one negative amplification control. The assays were conducted on an ABI 7500 fast system (ThermoFisher) using the following cycling conditions: 1 cycle at 50°C for 5 min; 1 cycle at 95°C for 10:00 min; and 40 cycles of 95°C for 3 s and 60°C for 30 s. Data were analyzed with a threshold setting at 5% maximum of the positive amplification control and 10% maximum of the positive internal control using design and analysis software Version 2.7. Primer and probe sequences are available upon request. Cq values ≥35 were considered negative.

### 2.3. Evaluation of Pathological Changes and Respiratory Tract Lesion Scoring

All lungs were independently scored for gross lesions by three pathologists using a previously described method [19]. Hematoxylin & eosin (H&E)‐stained slides were created for the examination of all collected fixed tissues and evaluated for lesions by a veterinary anatomic pathologist, anonymized to pig inoculation status. Sections of trachea and bronchi (5 µm thickness) from all pigs euthanized at 5 and 8 DPI were scored for lesions using a previously described scoring system for IAV by an anatomic pathologist anonymized to pig inoculation status [20]. Epithelial attenuation scores were defined as normal (0), focal cilia loss or flattening (1), mild focal to multifocal segmental loss of cilia with epithelial flattening (2), moderate multifocal to segmental loss of cilia with epithelial flattening (3), and severe segmental to diffuse loss of cilia with epithelial flattening (4). Cellular infiltration scores were defined as no infiltration (0), minimal infiltration (1), mild multifocal infiltration (2), moderate multifocal to coalescing infiltration (3), and severe diffuse infiltration (4). Each section of trachea and bronchus received a score of 0–4 (0 = normal, 4 = severe) for both cellular infiltration and epithelial attenuation, then added together for a total possible score range between 0 and 8. Histologic evaluation of the remaining collected fixed tissues, including fixed brain, lymph nodes, nasal turbinates, ethmoid, tonsils, liver, spleen, kidney, heart, and ileum, was also performed.

### 2.4. CD3 and CD20 IHC and QuPath Analysis

Sections of trachea and bronchi (4 μm) from all pigs that were euthanized at 5 and 8 DPI were stained for CD3 and CD20. Staining was performed on the Roche Diagnostics DISCOVERY ULTRA IHC/ISH research platform. Agilent’s CD3 (A0452, dilution 1:200) rabbit polyclonal antibody was used for T cell detection. Biocare’s CD20 rabbit polyclonal antibody (ACR 3004 B—Fisher NC1066362, dilution 1:100) was used for identification of B cells. All antibody incubations were 1 h in length. Roche’s DISCOVERY anti‐Rabbit HQ, anti‐HQ HRP, and ChromoMap DAB Kit were used for colorogenic signals with each antibody, followed by counterstaining the slides with hematoxylin.

Quantification of lymphocytes within the respiratory mucosa was performed using the QuPath image analysis platform (0.4.3) [21]. All slide preparations were digitized by an Aperio GT 450 slide scanner (Leica Biosystems, Deer Park, IL). Regions of interest in the trachea and bronchi, including the epithelium and lamina propria, were manually annotated. Submucosal glands, cartilage, and adventitia were not included within annotated regions. The number of lymphocytes within the mucosa was quantified by identifying the total number of IHC‐positive cells and then dividing by the total surface area of the annotated regions.

### 2.5. PoAstV4 RNAscope ISH and QuPath Analysis

Detection of PoAstV4 RNA in sections of trachea and bronchi from pigs euthanized at 5 DPI was performed with RNAscope with a probe targeting ORF1a (ACD catalog #530019). The following positive and negative control probes were used (SsPPIB catalog #428599 and dapB #312039). Staining was performed using the protease‐automated RNAscope VS Universal AP Assay for the Ventana DISCOVERY ULTRA System (Document Number UM 323250) with a deviation of 16 min for cell conditioning and 24 min for AMP 5.

Quantification of PoAstV4 ISH detection was performed with QuPath image analysis, as described for IHCs with the following modifications. The area of ISH staining within the epithelium was quantified and then divided by the total tissue area of the annotated regions, yielding percentages of positively stained areas. Only epithelium, excluding underlying lamina propria, was included within annotated regions.

### 2.6. PoAstV4 IgM and IgG Enzyme‐Linked Immunosorbent Assays (ELISAs)

Serum ELISAs were performed to evaluate both serum IgM and IgG responses against the recombinant PoAstV4 capsid spike antigen (GenBank #PP806170.1, amino acids 420–655) using an adapted protocol [22]. Three 96‐well medium‐binding ELISA plates (Corning #9017) were coated with 50 µL of 10 µg/mL purified recombinant PoAstV4 capsid spike in phosphate‐buffered saline pH 7.4 (PBS), or PBS alone as a “no antigen” control, covered with microplate sealing tape (Corning #6575), and incubated overnight at 4°C. The plate was washed three times (200 μL each) with PBS‐T (PBS + 0.1% Tween). Then, 200 μL of blocking solution (PBS‐T + 5% milk) was added to all wells of the plate and incubated for 2 h at room temperature. After the incubation, the blocking solution was removed and tapped on a Kimwipe to dry. Next, 396 μL of blocking buffer and 4 μL of inoculated or negative control porcine serum were added to a 96‐well mixing block and mixed with a multichannel pipette. Then, 120 μL of the diluted serum was transferred from its corresponding well in the fraction collector to the same well on the ELISA plate, making a 1:100 dilution on the plate. Each serum sample was added in triplicate, with three columns dedicated to an individual sample. The plate was incubated for 2 h at room temperature and then washed 3 times with 200 µL PBS‐T. Next, 50 µL of either anti‐swine IgG‐HRP secondary antibody (Jackson ImmunoResearch Laboratories #114‐035‐003) diluted at 1:10,000 in PBS‐T + 1% milk or anti‐swine IgM‐HRP secondary antibody (BioRad #AAI48P) diluted at 1:3000 in PBS‐T + 1% milk was added to each well, and the plate was incubated for 1 h at room temperature. The plate was then washed three times with 200 µL PBS‐T. To develop the plate, 100 µL of TMB substrate (Sigma #T0440) was added to all wells and incubated for 8 min at room temperature, followed immediately by quenching with 100 µL 1 N H_2_SO_4_. The 450 nm absorbance in each well was measured with a plate reader.

### 2.7. Statistical Analysis

Data were analyzed with GraphPad Prism 10.2.3. Comparisons between means were first evaluated with two‐way ANOVA tests followed by unpaired *T* tests with Welch’s correction to evaluate statistical significance. Spearman correlations were used to compare histological lesions and ISH‐stained areas in the trachea and bronchi.

## 3. Results

### 3.1. PoAstV4 Detection in the Respiratory Tract and Rectal Swabs

**Figure 2:**
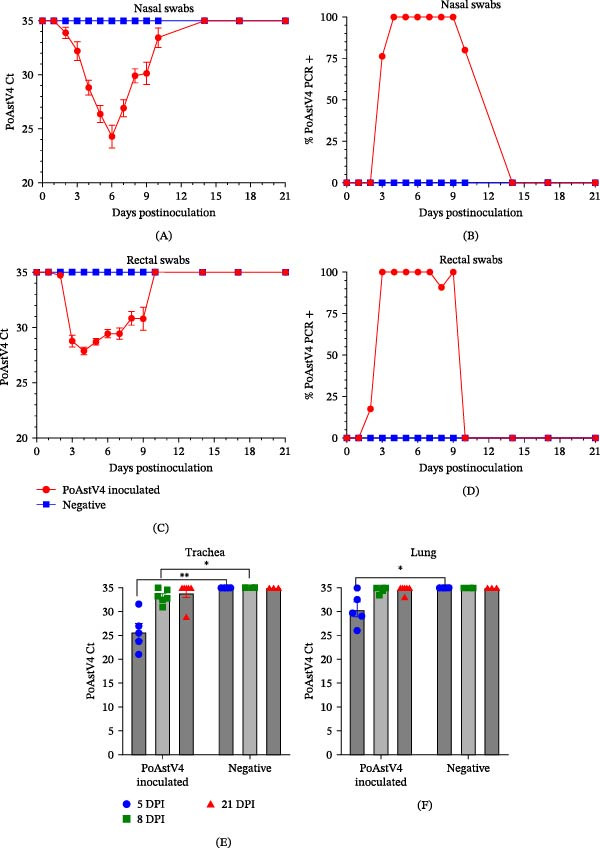
PoAstV4 detection in swabs and respiratory tissues via RT‐qPCR. Nasal shedding of PoAstV4, including the percentage of pigs that tested positive in both inoculated and negative groups over time (A, B). Rectal shedding of PoAstV4, including the percentage of pigs that tested positive in both inoculated and negative groups over time (C, D). Presence of PoAstV4 within respiratory tissues was evaluated with PoAstV4 PCR at 5, 8, and 21 DPI. Data for graphs (A, C, E, and F) are presented as means with standard error mean bars. In graphs (E, F), data for individual animals are presented as individual symbols. *p* < 0.05 were considered significant ( ^∗^ < 0.05,  ^∗∗^ < 0.01).

At 2 DPI, PoAstV4 was detected in nasal swabs from 6 of 17 inoculated pigs at high Ct values (>27) (Figure 2A). By 4 DPI, all 17 inoculated pigs were positive for PoAstV4 via nasal swabs (Figure 2B). Peak detection of the virus occurred at 6 DPI with a gradual decrease in quantity of PoAstV4 nucleic acid and cessation of detection of PoAstV4 in nasal swabs by 14 DPI. Rectal swabs tested positive for PoAstV4 at 2 DPI in 3 of 17 pigs (Figure 2B). Peak detection of PoAstV4 in rectal swabs occurred at 4 DPI with an end of detection at 10 DPI (Figure 2C, D). The mean Ct value at which peak viral detection occurred in nasal swabs was 24.3 compared to 27.8 in rectal swabs, indicating increased detectable viral nucleic acid in nasal swabs compared to rectal swabs. Control pigs tested negative for PoAstV4 via qPCR in all nasal and rectal swabs and tissues throughout the study (Figure 2).

Nasal and fecal swabs with the lowest Ct values were sequenced at the ISU VDL. Comparison showed nearly identical whole genome sequences (>98.8% homology), indicating that the virus detected in the rectum is representative of the virus from the respiratory tract.

Trachea and lung sections collected at necropsies on 5, 8, and 21 DPI were tested for PoAstV4 via RT‐qPCR (Figure 2C, D). Peak detection of the virus in both tissue types was at 5 DPI with a large reduction in detected virus at 8 DPI. Only one inoculated pig had positive respiratory tissues on 21 DPI.

### 3.2. Evaluation of Microscopic Lesions and Detection of PoAstV4 Within Lesions via ISH

**Figure 3:**
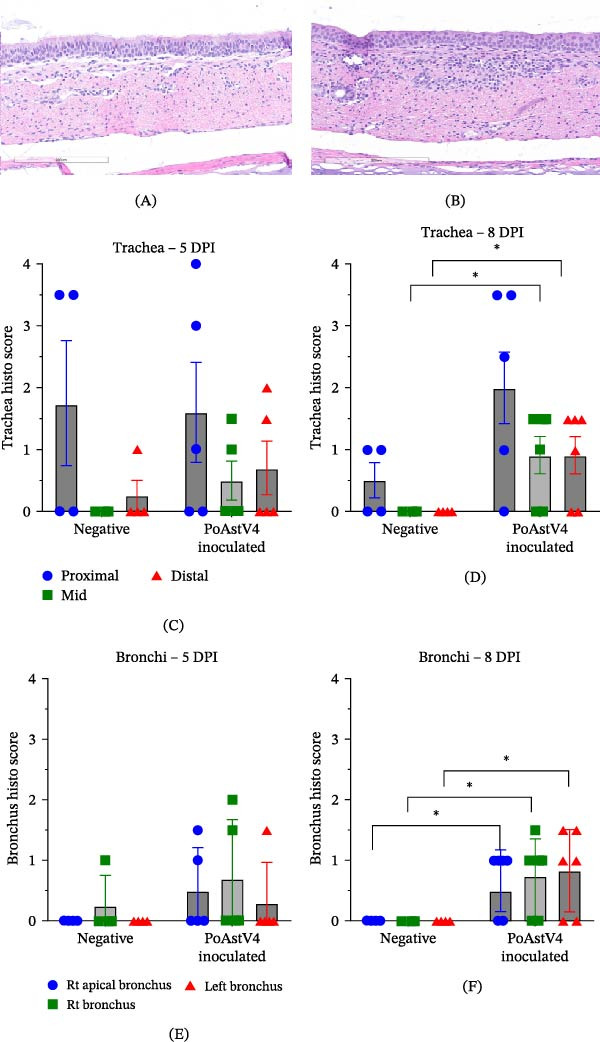
Microscopic lesions of tracheitis and bronchitis. Hematoxylin and eosin (H&E)‐stained slides display tracheal tissue from negative control (A) and PoAstV4‐inoculated pigs (B) at 5 DPI. Inoculated pigs had increased numbers of lymphocytes within the lamina propria with attenuation of the epithelium and loss of cilia (B) compared to negative controls (A). Sections of trachea and all three mainstem bronchi from pigs euthanized at 5 and 8 DPI were evaluated for histologic lesions affecting the epithelium and/or the infiltration of lymphocytes into the mucosa (C–F). Quantified data are presented as means with standard error mean bars, with data from individual animals represented by individual symbols. *p*‐Values were considered significant ( ^∗^ < 0.05).

Sections of trachea were evaluated at three distinct regions (proximal trachea, mid trachea, and distal trachea) to both control for intubation‐related lesions, as well as to provide insight for sample collection in cases of diagnostic investigation. At 5 DPI, the proximal trachea of both negative control and PoAstV4‐challenged pigs had the most severe lesions of tracheal epithelial attenuation and mucosal cellular infiltration (Figure 3A–C). However, considering that intratracheal inoculation was performed via intubation, this was not surprising and could be attributed to mucosal injury from intubation. At 8 DPI, histologic scores were statistically higher in the mid and distal trachea for inoculated pigs compared to controls (Figure 3D). At 5 DPI, there was a numerical trend for higher bronchial lesion scores in inoculated pigs, but these were not statistically significant when compared to control pigs (Figure 3E). However, by 8 DPI, there were more severe histologic lesions observed in all three bronchi collected from the PoAstV4‐inoculated pigs (Figure 3F). Lungs were also evaluated at all time points. Two pigs, one within the negative control group and one within the inoculated group, displayed increased alveolar neutrophils and macrophages consistent with mild bronchopneumonia. The remaining lung sections were unremarkable, with no lesions of bronchiolitis or secondary bacterial pneumonia.

**Figure 4:**
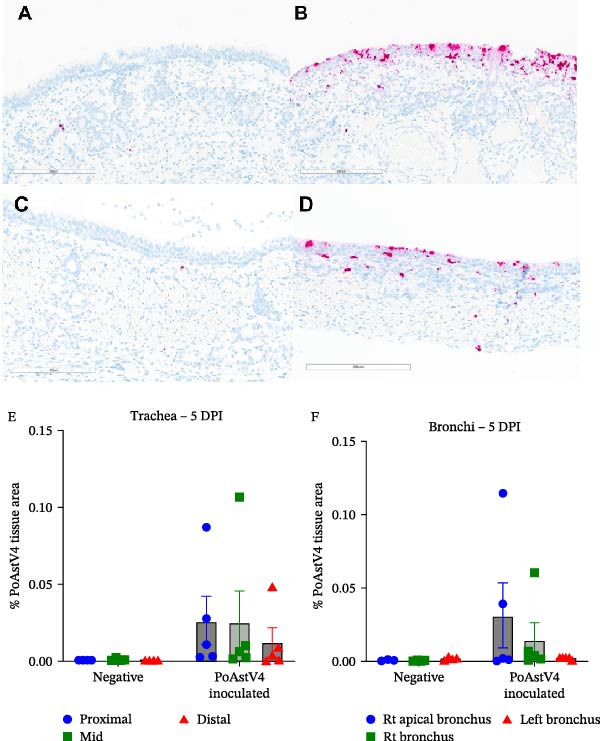
Localization of virus within lesions via RNAscope ISH at 5 DPI. Nasal turbinates: PoAstV4 ISH staining (red) in negative control and PoAstV4‐inoculated pigs (A, B). The inoculated pig has red staining (detection of viral RNA) within the mucosa. Trachea: PoAstV4 ISH staining (red) in negative control and PoAstV4‐inoculated pigs (C, D). PoAstV4 ISH staining of the trachea and bronchi at 5 DPI, presented as positive tissue area divided by the evaluated tissue area in pigs euthanized on 5 DPI (E, F). Quantified data are presented as means with standard error mean bars with data from individual animals represented by individual symbols. Scale bars = 200 μm.

PoAstV4 ISH was performed on nasal turbinates collected from animals euthanized at 5 DPI. There was abundant staining in the epithelium of evaluated sections in inoculated pigs (Figure 4B). Histological lesions of epithelial attenuation or inflammation were not evaluated in turbinates, as repeated nasal swab sampling and subsequent traumatic injury would have confounded results. PoAstV4 was detected in the mucosa of inoculated pigs with RNAscope ISH (Figure 4D). While inoculated pigs had numerically higher amounts of PoAstV4 ISH‐stained area within the mucosa of both the trachea and bronchi, it was not statistically significant when compared to negative control pigs (Figure 4E, F). However, there was a significant positive correlation of PoAstV4 ISH staining with histological lesion scores in the trachea (*r* = 0.700 *p* = 0.005) and a nearly significant positive correlation in the bronchi (*r* = 0.517 *p* = 0.051).

### 3.3. Lymphocytic Respiratory Mucosa Infiltration

**Figure 5:**
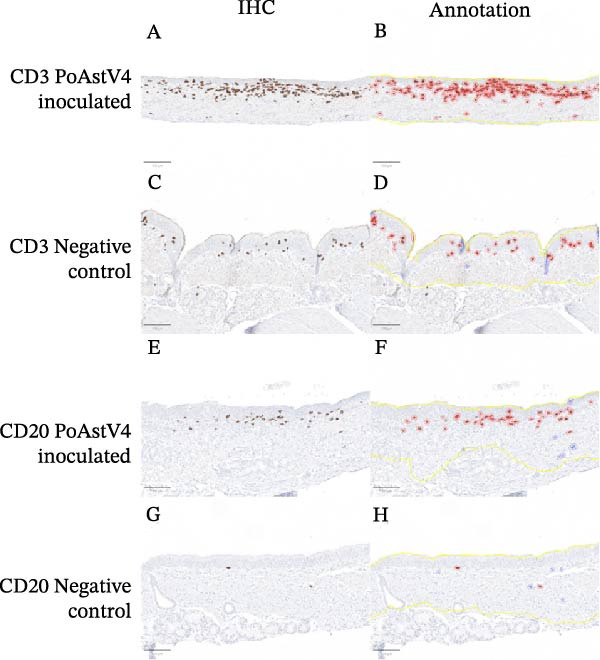
Analysis of lymphocytic infiltration into tracheal mucosa. CD3 IHC (A, C) with the detection of T cells with QuPath software and identification of individual cells in red (B, D). The evaluated mucosa is delineated by the yellow lines. CD20 IHC (E, G) with the detection of B cells with QuPath software and identification of individual cells in red (F, H). The evaluated mucosa is delineated by the yellow lines.

**Figure 6:**
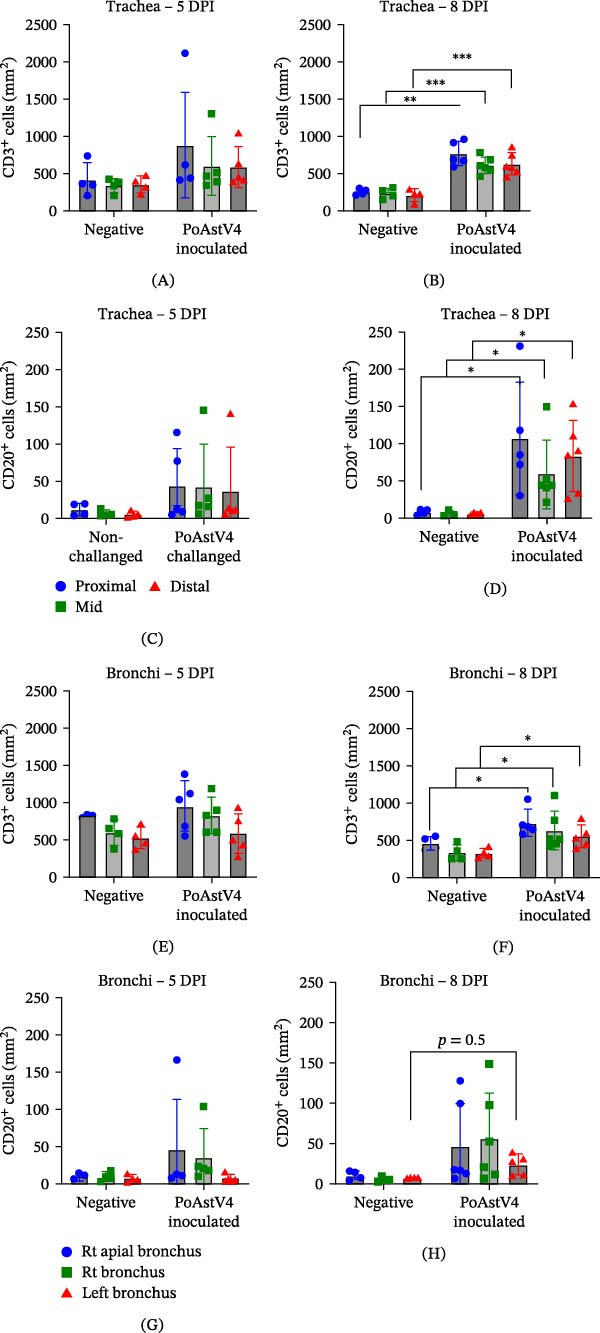
Quantification of lymphocytic infiltration of respiratory mucosa. T cell infiltration of the tracheal mucosa at 5 and 8 DPI was evaluated with CD3 IHCs quantified with QuPath as number of cells/area mm^2^ (A, B). B cell infiltration of the tracheal mucosa was evaluated with CD20 IHCs quantified with QuPath as number of cells/area evaluated (C, D). Bronchi were similarly assessed for T and B cell infiltration at 5 and 8 DPI (E–H). Data are presented as means and standard deviation error bars, with data from individual animals represented by individual symbols. *p*‐Values were considered significant ( ^∗^ < 0.05,  ^∗∗^ < 0.01,  ^∗∗∗^ < 0.001).

The infiltration of respiratory mucosa by lymphocytes is one of the key features of lesions of respiratory epitheliotropic viral infection [23, 24]. Therefore, T (CD3^+^) and B (CD20^+^) cells were identified with IHCs and quantified with image analysis software (QuPath) by dividing the number of IHC‐positive cells within the mucosa by the total area of mucosa evaluated (Figure 5). While inoculated pigs had numerically higher numbers of both T and B cells in the mucosa of the trachea and bronchi at 5 DPI, results were not statistically significant when compared to control pigs (Figure 6A, C, E, G). However, by 8 DPI, inoculated pigs had significantly more T and B cells in the mucosa of the trachea as well as more T cells in the mucosa of all three bronchi (Figure 5B, D, F). While B cell levels in the bronchial mucosa were not statistically significant, they were numerically higher, and statistical comparison of inoculated and negative control pigs showed a *p*‐value of 0.05 for the left bronchus (Figure 5H).

### 3.4. PoAstV4 IgM and IgG Seroconversion

**Figure 7:**
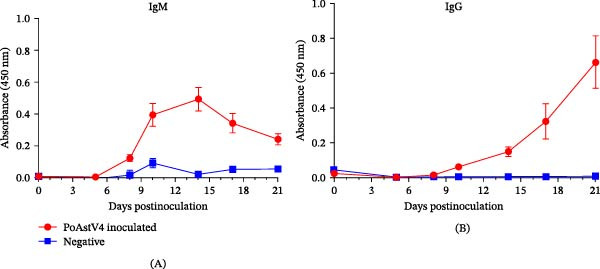
PoAstV4 spike IgM and IgG seroconversion. All results are presented as optical density. The 5 and 8 DPI results are from pigs that were euthanized on those days. Results from 10 to 21 DPI are from the same animals (PoAstV4‐inoculated *n* = 5, negative *n* = 3). Data are presented as means with standard mean error bars.

PoAstV4 IgM and IgG seroconversion were evaluated using a PoAstV4 capsid spike ELISA (20). Antibodies against IgM became detectable at 8 DPI with a peak of response at 14 DPI and contraction thereafter. IgG antibodies against PoAstV4 were first detectable at 10 DPI with a steady increase in quantity up to the end of the study at 21 DPI. All sera were negative for anti‐PoAstV4 IgM and IgG on 0 DPI (Figure 7).

## 4. Discussion

This research shows an association between PoAstV4 infection and both tracheitis and bronchitis in young growing pigs. Reproduced microscopic lesions were not as severe, nor as low in the respiratory tract, as has previously been observed in some PoAstV4 naturally infected cases [15]. Microscopic lesion severity may have been impacted by both the amount of infectious PoAstV4 in the inoculum as well as any host components in the lung homogenate, such as inflammatory mediators and/or cellular debris that could have caused varying inflammatory responses. Filtered respiratory tissue homogenate was used for inoculation due to the current inability to isolate PoAstV4. Astroviruses are notoriously difficult to isolate; thus, reproduction of disease studies are often performed with tissue homogenates that have been evaluated to ensure that no other potential primary pathogens are present [25, 26]. In this case, a matched control lung homogenate from a commercial pig free of any respiratory pathogens was not feasible. The inoculum was screened via PCR to ensure that there was a low Ct value (19.2). Although without viral isolation, there is no way of knowing the quantity of infectious PoAstV4 in the inoculum. The lower viral loads (higher Ct values) observed in this experimental model, compared to field cases, may also reflect the high health status of CDCD pigs.

The identification of PoAstV4 rectal shedding is interesting considering previous reports of PoAstV4 in feces [14, 27, 28]. The timing, amount of virus, and the genetic homology between the virus detected in the nasal and fecal swabs suggest that the PoAstV4 detected in feces may be virus that was expelled from the respiratory tract and subsequently may have been swallowed. However, it is also possible that the comparatively lower level of viral RNA in feces may be due to dilution in fecal material. While PoAstV4 was localized to the respiratory tract in this study, the virus inoculum may have infected the gastrointestinal tract as well. However, gross and histologic evaluation of the ileum were unremarkable. Additionally, PoAstV4 ISH performed on the ileum was negative for pigs at 5 DPI. As the focus of this study was on the respiratory tract, the duodenum, jejunum, stomach, and colon were not evaluated and could be sites of replication based on other published studies of different astroviruses and the ability of human astroviruses to replicate in a colon carcinoma cell line (Caco‐2) [18, 26, 29–31].

The immune response to PoAstV4 infection has not been previously described. There is both a productive antibody and cell‐mediated immune response, as evidenced by IgM and IgG seroconversion and the identification of cellular infiltration into affected tissues in inoculated pigs. The serum IgM and IgG dynamics appear to match previous findings with PoAstV3 [12]. The inability to isolate the virus currently prevents the evaluation of serum neutralizing antibodies; however, studies of antibodies against human astrovirus capsid core and spike domains reveal that only antibodies to the capsid spike are neutralizing [32]. As the ELISA in this study evaluated antibodies against the PoAstV4 capsid spike, it is suspected that neutralizing antibodies were elicited. At 8 DPI, the quantification of the density of T cells within respiratory tract mucosa showed a cellular immune response in inoculated vs. negative control pigs. Future investigations into the antigen‐specific response and phenotypes of T cell populations would be foundational in understanding the importance of the T cell response for immune protection from PoAstV4 disease.

## 5. Conclusion

After challenge, PoAstV4 infected nasal, tracheal, and bronchial epithelium and was shed in nasal secretions for approximately 10 days with a peak at 6 DPI. Histological and seroconversion data indicate that there is both a cell‐mediated immune response, dominated by CD3^+^ T cells, and a standard IgM and IgG antibody response to infection. Future efforts should be directed towards genetic characterization and isolation of the virus so that additional studies fulfilling Koch’s postulates and evaluating the impact of PoAstV4 on pig growth and coinfections can be performed.

## Funding

This work was supported by a grant from the Swine Health Information Center (SHIC) (Project Number #23‐077).

## Disclosure

All opinions in the article are the authors’ and do not necessarily reflect the policies and views of USDA. Mention of trade names or products is for informational purposes only and does not imply endorsement by the USDA. USDA is an equal opportunity employer and provider.

## Conflicts of Interest

The authors declare no conflicts of interest.

## Data Availability

The data that support the findings of this study are available from the corresponding author upon reasonable request.
